# Forensic Age Estimation Using Tooth Eruption Status in Children: A Correlative Study With Chronological Age

**DOI:** 10.7759/cureus.107010

**Published:** 2026-04-14

**Authors:** Sreenitha S Hosthor, Sreelatha S Hosthor, Abdul Habeeb B Mohsin, Rahul Tiwari, Padmanabhuni Kalyani, Rahul Anand, Manish Sharma

**Affiliations:** 1 Forensic Odontology, Government Dental College and Research Institute Bengaluru, Bengaluru, IND; 2 Oral Pathology, Krishnadevaraya College of Dental Sciences and Hospital, Bengaluru, IND; 3 Prosthodontics, Military Medical City Hospital, Ar-Rayyan, QAT; 4 Oral and Maxillofacial Surgery, Narsinhbhai Patel Dental College and Hospital, Sankalchand Patel University, Visnagar, IND; 5 Oral Pathology, Care Dental College and Hospital, Guntur, IND; 6 Oral and Maxillofacial Surgery, Late Shri Yashwantrao Chavan Memorial Medical and Rural Development Foundation's Dental College and Hospital, Ahilyanagar, IND; 7 Oral Pathology, Jawahar Medical Foundation's Annasaheb Chudaman Patil Memorial Dental College, Dhule, IND

**Keywords:** age estimation, children, forensic dentistry, mixed dentition, tooth eruption

## Abstract

Introduction: Forensic age estimation is essential in clinical and legal settings, particularly in children, where reliable and non-invasive methods are required. Tooth eruption follows a relatively consistent developmental pattern and may serve as a practical indicator of chronological age during the mixed-dentition period. This study aimed to evaluate the relationship between cumulative tooth eruption status and chronological age in children and to develop a predictive model for age estimation.

Materials and methods: A cross-sectional observational study was conducted on 120 children aged 6-13 years in the Department of Oral Pathology at Jawahar Medical Foundation's Annasaheb Chudaman Patil Memorial Dental College, Dhule. Chronological age was calculated in decimal years using verified birth records. Clinical examination of 28 permanent teeth (excluding third molars) was performed, and eruption status was scored as 0 (unerupted), 1 (partially erupted), or 2 (fully erupted), with cumulative scores calculated for each participant. Data were statistically analyzed. Normality was assessed using the Shapiro-Wilk test. Independent samples t-test, one-way analysis of variance (ANOVA) with Tukey’s post-hoc analysis, Pearson’s correlation, and linear regression were applied. Statistical significance was set at P < 0.05.

Results: The mean chronological age was 9.8 ± 2.3 years, and the mean eruption score was 32.6 ± 8.4. No significant sex differences were observed in age (p = 0.134) or eruption score (p = 0.174). A significant increase in eruption score across age groups was observed (p < 0.001). Post-hoc analysis confirmed significant differences among all age groups (p < 0.05). A strong positive correlation was observed between the eruption score and chronological age (r = 0.72, p < 0.001). Simple linear regression analysis demonstrated that the eruption score significantly predicted age (R² = 0.62, p < 0.001).

Conclusion: Cumulative tooth eruption status shows a strong association with chronological age and serves as a reliable, non-invasive indicator for age estimation in children, with good discriminatory ability and practical applicability in clinical and forensic settings.

## Introduction

Forensic age estimation plays a crucial role in human identification, particularly in situations involving unknown individuals, mass disasters, child labor cases, and legal disputes concerning age. Among various biological indicators, dental parameters are considered one of the most reliable tools for age estimation because of their relative resistance to environmental influences and postmortem changes [1,2]. Teeth exhibit a well-defined pattern of growth and development, making them valuable markers for forensic investigations, especially in pediatric populations where skeletal maturity assessment may show variability [3].

Dental age estimation methods can be broadly categorized into radiographic and clinical approaches. Radiographic techniques, such as those based on tooth mineralization stages, are widely regarded as more accurate; however, they require specialized equipment, expose individuals to radiation, and may not be feasible in all settings [4]. In contrast, clinical methods based on tooth eruption status offer a simple, noninvasive, and cost-effective alternative. Tooth eruption follows a relatively consistent chronological sequence, though it may be influenced by factors such as genetics, nutrition, and systemic health [5]. Despite these variations, eruption-based assessment remains a practical tool, particularly for large-scale or resource-limited forensic scenarios.

In children, the mixed dentition period provides an ideal window for evaluating eruption patterns as multiple permanent teeth emerge during this phase. Establishing a correlation between tooth eruption status and chronological age can contribute to the development of population-specific standards, which are essential for improving the accuracy of forensic age estimations [6]. Such data are particularly important in diverse populations where existing standards may not be directly applicable.

The aim of the present study was to evaluate the relationship between tooth eruption status and chronological age in children, and to assess its applicability in forensic age estimation. The objectives were to determine the correlation between eruption score and chronological age, develop a predictive model for age estimation based on eruption status, and analyze potential differences in eruption patterns between sexes.

## Materials and methods

This cross-sectional observational study was conducted at Jawahar Medical Foundation’s Annasaheb Chudaman Patil Memorial Dental College, Dhule, India, from June 2023 to December 2023. Ethical approval (EC/NEW/INST/2022/2959/2023/02/SS12) was obtained from the Institutional Ethics Committee prior to the commencement of the study. The study involved only noninvasive intraoral examination without radiographic or photographic procedures, thereby posing minimal risk to participants. Written informed consent was obtained from the parents or guardians, and verbal consent was obtained from all participating children.

The sample size was calculated based on the expected correlation between tooth eruption status and chronological age derived from previous literature (r ≈ 0.6) [7]. Using a two-tailed test with a significance level (α) of 0.05 and statistical power of 80%, the minimum required sample size was estimated using a correlation coefficient formula of 85 participants. To enhance the robustness and generalizability of the findings and account for potential variability, a total of 120 children were included in the study.

Children aged 6-13 years were recruited from the outpatient department using a convenience sampling technique. This age group corresponds to the mixed dentition period, during which active eruption of permanent teeth occurs, making it suitable for eruption-based age estimations. Because a non-probability sampling method was employed, the findings should be interpreted with caution in terms of generalizability.

Only children with a verified date of birth, confirmed through official records such as birth certificates or school documents, were included. The participants were required to be systemically healthy and free from developmental dental anomalies. Children with a history of orthodontic treatment, craniofacial anomalies, systemic diseases affecting growth and development, or altered eruption patterns due to premature loss or prolonged retention of the primary teeth were excluded. Additionally, participants presenting with impacted teeth, supernumerary teeth, or pathological conditions affecting tooth eruption were excluded.

Chronological age was calculated by subtracting the date of birth from the date of clinical examination, and was expressed in decimal years to improve precision and facilitate statistical analysis. A comprehensive intraoral examination was performed under standard dental operatory illumination using a plane mouth mirror and a CPI probe, following strict infection control protocols. All permanent teeth, excluding the third molars (a total of 28 teeth), were assessed clinically.

The tooth eruption status was evaluated using a standardized scoring (self-validated) system. A score of 0 was assigned to a non-erupted tooth, a score of 1 to a partially erupted tooth with a visible crown structure but not in functional occlusion, and a score of 2 to a fully erupted tooth in functional occlusion. The individual scores for all the assessed teeth were summed to obtain a cumulative eruption score for each participant, with a maximum possible score of 56. This simplified scoring system was adopted to provide a practical and clinically applicable measure of the eruption status.

All clinical examinations were performed by a single trained examiner to eliminate interexaminer variability. Prior to the main study, an intraexaminer calibration was conducted on 20 children who were not included in the final sample. Each subject was examined twice at a one-week interval. Intraexaminer reliability was assessed using Cohen’s kappa statistic, and a value greater than 0.85 was considered indicative of excellent agreement and reproducibility. The primary outcome measure was the correlation between the cumulative eruption score and chronological age. Secondary outcomes included evaluation of eruption patterns across different age groups and assessment of gender-based differences in eruption timing.

Data were entered into Microsoft Excel (Microsoft Corporation, USA) and subsequently analyzed using IBM Statistical Package for the Social Sciences (SPSS) Statistics (version 25.0, IBM Corp., USA). Descriptive statistics, including mean and SD, were calculated for chronological age and eruption scores. The normality of the data distribution was assessed using the Shapiro-Wilk test. As the data followed a normal distribution, Pearson’s correlation coefficient was applied to evaluate the relationship between eruption score and chronological age. Simple linear regression analysis was performed with chronological age as the dependent variable and the cumulative eruption score as the independent variable to derive a predictive equation for age estimation. The assumptions of linearity, independence, and homoscedasticity were verified prior to model application. An independent samples t-test was used to compare eruption scores between male and female participants. The equality of variances was assessed using Levene’s test before applying it. Statistical significance was set at P < 0.05.

## Results

A total of 120 children aged 6-13 years were included in the study. The distribution of participants across age groups and sex is presented in Table 1, demonstrating adequate representation of the mixed dentition period with a balanced sex distribution.

**Table 1:** Distribution of study participants according to age group and sex (N = 120) Data are presented as number (percentage).

Variable	Category	n (%)
Age group	6–7 years	20 (16.7)
	8–9 years	25 (20.8)
	10–11 years	35 (29.2)
	12–13 years	40 (33.3)
Sex	Male	62 (51.7)
	Female	58 (48.3)

Descriptive statistics for the chronological age and cumulative eruption scores are summarized in Table 2. The overall mean chronological age of the study population was 9.8 ± 2.3 years, while the mean cumulative eruption score was 32.6 ± 8.4. Both variables exhibited a broad range, reflecting variability in eruption timing within the studied age span. No statistically significant differences were observed between male and female participants for either chronological age (p = 0.134) or eruption score (p = 0.174), indicating comparable eruption patterns across sexes.

**Table 2:** Descriptive statistics of chronological age and cumulative eruption score Data are presented as mean ± SD; Independent t-test used for comparison between male and female participants; p > 0.05 denotes no statistical significance

Variable	Category	Mean ± SD	Minimum	Maximum	t value	p-value
Chronological age (years)	Overall	9.8 ± 2.3	6.0	13.0	1.50	0.134
	Male	10.1 ± 2.6	6.0	13.0		
	Female	9.3 ± 3.2	6.0	13.0		
Total eruption score	Overall	32.6 ± 8.4	12	48	1.36	0.174
	Male	31.1 ± 8.6	12	48		
	Female	33.2 ± 8.2	12	46		

**Figure 1:**
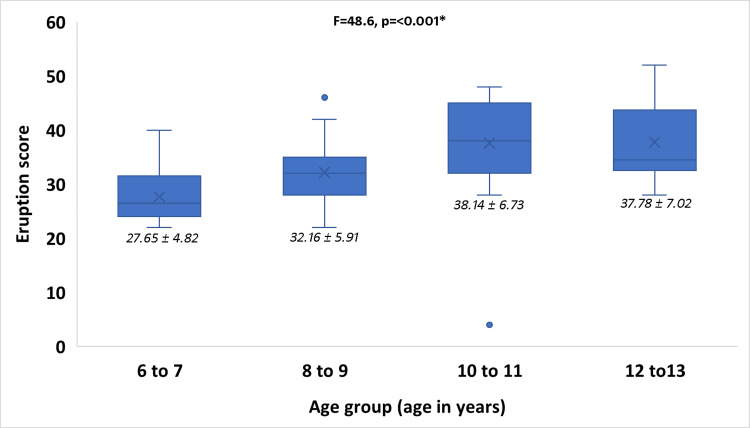
Mean eruption score across age groups Bar graph illustrating the mean and SD of cumulative eruption score across different age groups (6–7, 8–9, 10–11, and 12–13 years). *One-way ANOVA analysis showed significant difference between age groups (F = 48.6, p < 0.001). ANOVA: Analysis of variance

The distribution of eruption scores across the different age groups showed a clear increasing trend, as illustrated in Figure 1. One-way analysis of variance (ANOVA) demonstrated a statistically significant difference in mean eruption scores among the age groups (p < 0.001), indicating that eruption status varies significantly with advancing age.

Post-hoc analysis using Tukey’s test (Table 3) confirmed that all pairwise comparisons between age groups were statistically significant (p < 0.05), with progressively higher eruption scores observed in the older age groups. This consistent hierarchical progression supports the validity of the cumulative eruption score as a discriminative marker of developmental stage within the 6-13 year age range.

**Table 3:** Post-hoc comparison of mean eruption scores between age groups using Tukey's test Tukey post hoc test applied following one-way ANOVA. *Statistically significant (p < 0.05) CI: Confidence interval; ANOVA: Analysis of variance

Pairwise age group comparison	Mean difference	Standard error	t value	p-value	95% CI lower limit	95% CI upper limit
6-7 vs. 8-9	-4.7	1.56	-3.02	0.019*	-8.88	-0.52
6-7 vs. 10-11	-11.93	1.45	-8.21	< .001*	-15.83	-8.03
6-7 vs. 12-13	-17.95	1.42	-12.64	< .001*	-21.76	-14.14
8-9 vs. 10-11	-7.23	1.36	-5.32	< .001*	-10.87	-3.58
8-9 vs. 12-13	-13.25	1.32	-10.02	< .001*	-16.8	-9.7
10-11 vs. 12-13	-6.02	1.2	-5.02	< .001*	-9.24	-2.8

**Figure 2:**
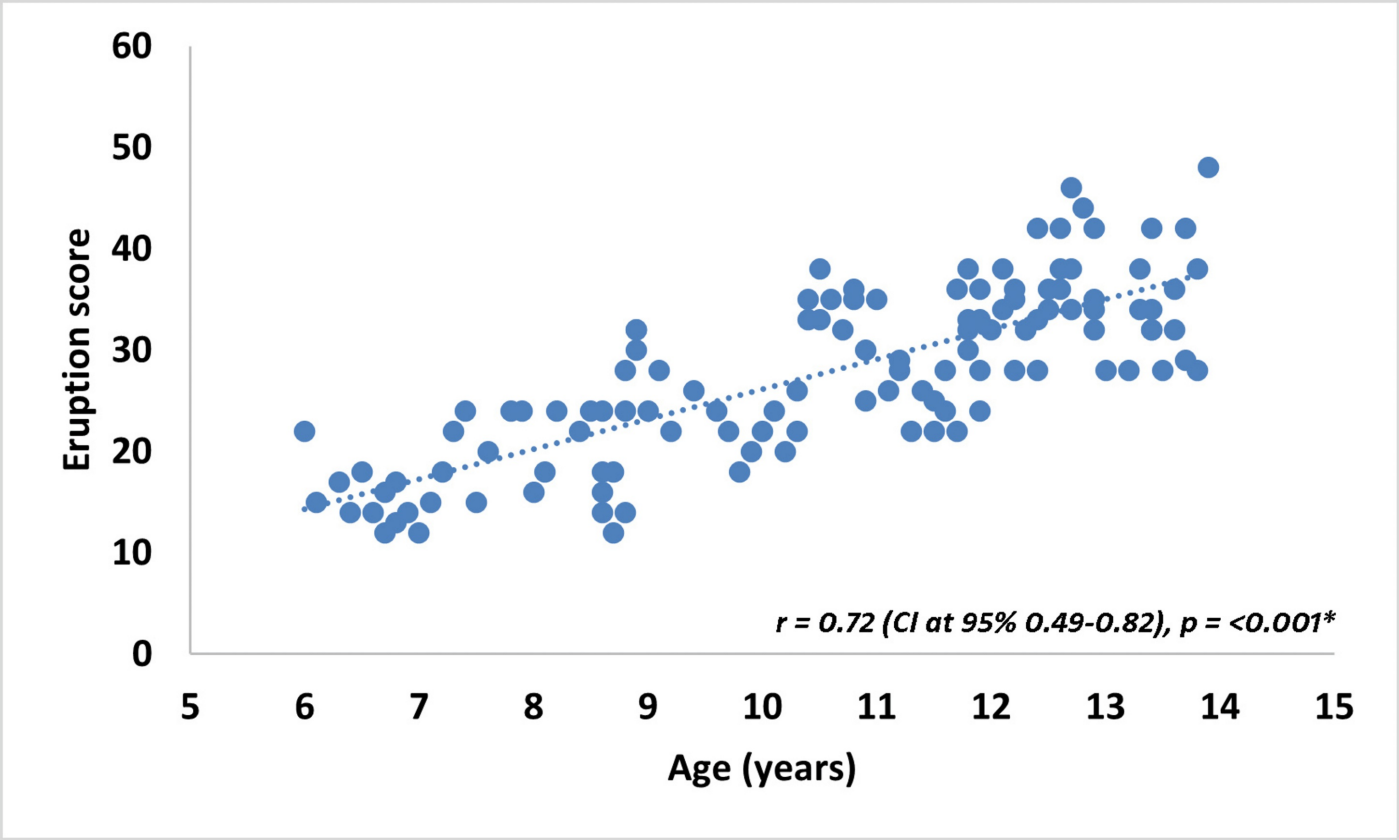
Pearson's correlation between chronological age and cumulative eruption score Scatter plot showing the relationship between chronological age and cumulative eruption score; the plot demonstrates a strong positive linear correlation, with increasing eruption scores corresponding to advancing chronological age. *p < 0.05 significant CI: Confidence interval

Correlation analysis revealed a strong positive relationship between chronological age and cumulative eruption score (Figure 2), with Pearson’s correlation coefficient of r = 0.72 (p < 0.001). This indicates that approximately 72% of the variability in eruption scores is linearly associated with chronological age, reflecting a robust biological relationship between dental eruption and age.

Simple linear regression analysis (Table 4) demonstrated that the cumulative eruption score was a significant predictor of the chronological age. The regression model was statistically significant (p < 0.001), and explained 62% of the variance in age (R² = 0.62).

**Table 4:** Simple linear regression analysis for prediction of chronological age based on eruption score Dependent variable: Chronological age; Independent variable: Cumulative eruption score; Model fit: R² = 0.62 *Statistically significant (p < 0.05) CI: Confidence interval

Model	β coefficient	Standard error	t value	p-value	95% CI of β coefficient
Constant	4.70	0.44	10.78	< 0.001*	3.84 – 5.58
Eruption score	0.21	0.02	14.01	< 0.001*	0.18 -0.24

The derived regression equation is as follows: 

\[ \text{Age} = 4.70 + 0.21 \times \text{Eruption score} \]

In this equation, the constant (intercept) is 4.70, representing the estimated age (in years) when the eruption score is zero. The regression coefficient (slope) for eruption score is 0.21 (β = 0.21), indicating that for each one‑unit increase in the eruption score, the predicted age increases by 0.21 years (approximately 2.5 months). The regression coefficient for eruption score was highly significant (β = 0.21, p < 0.001; 95% confidence interval (CI): 0.18-0.24), confirming its strong predictive value.

**Figure 3:**
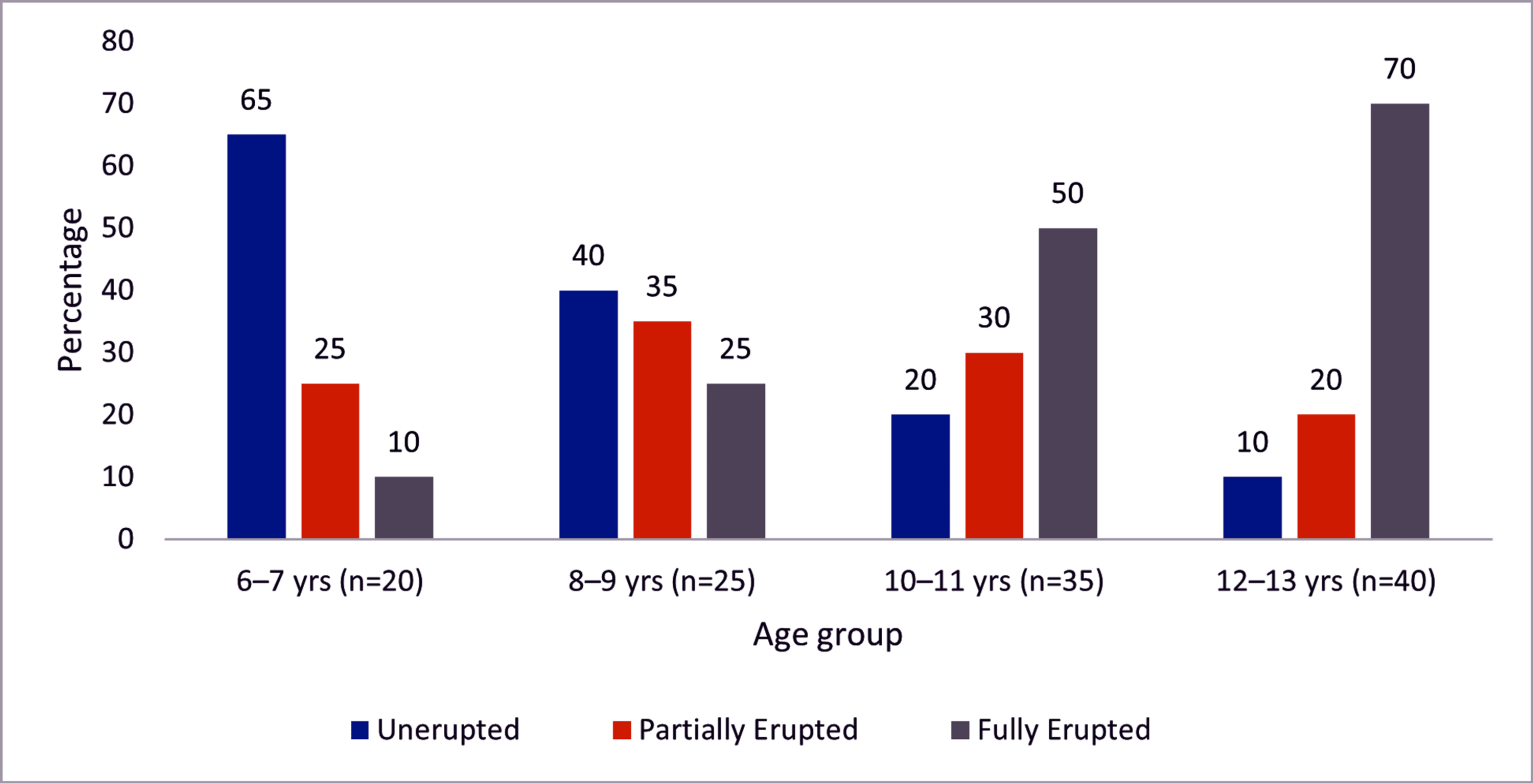
Age-wise distribution of tooth eruption status Bar graph depicting the percentage (%) distribution of unerupted, partially erupted, and fully erupted teeth across different age groups.

The age-wise distribution of the tooth eruption status is illustrated in Figure 3, demonstrating a clear developmental transition with advancing age. Younger children predominantly exhibited unerupted teeth, whereas older children showed a marked increase in the number of fully erupted teeth. Partially erupted teeth were the most prevalent in the intermediate age groups, reflecting the transitional stages of dental development. This progressive shift further substantiates the biological consistency of eruption patterns and reinforces their applicability to age estimation.

Overall, the findings demonstrated a strong, statistically significant, and biologically consistent relationship between tooth eruption status and chronological age. The cumulative eruption score exhibited excellent discriminatory ability across age groups and strong predictive potential, supporting its use as a reliable, non-invasive tool for forensic age estimation in children.

## Discussion

The present study evaluated the relationship between cumulative tooth eruption status and chronological age in children aged 6-13 years, and demonstrated a strong, statistically significant association between the two variables. These findings confirm that tooth eruption follows a consistent and progressive pattern with advancing age and can serve as a reliable biological marker for age estimation in the mixed dentition period.

A key finding of this study was the strong positive correlation between the eruption score and chronological age (r = 0.72, p < 0.001), indicating that dental eruption closely parallels age progression. This is in agreement with previous studies that have reported significant correlations between eruption status and chronological age in pediatric populations. For instance, Pooja et al. reported a similar trend in South Indian children, highlighting that eruption patterns can be effectively used for age estimation in population-specific contexts [7]. Likewise, a study by Logan and Kronfeld has established the chronological sequence of tooth eruption as a dependable developmental indicator [8].

Thomas et al. demonstrated that the eruption status of mandibular premolars and second molars can serve as a reliable indicator for distinguishing whether an individual is above or below 14 years of age [9]. Their orthopantomographic analysis of South Indian children supports the forensic applicability of eruption-based dental markers for age estimation around critical legal thresholds. The present study also demonstrated a statistically significant difference in eruption scores across age groups, with a clear hierarchical progression from younger to older children. The results of the one-way ANOVA and Tukey post-hoc analysis confirmed that each age group exhibited significantly different eruption scores, reinforcing the discriminatory capacity of cumulative eruption scoring.

Linear regression analysis further strengthened these observations by demonstrating that the eruption score is a significant predictor of chronological age, explaining approximately 62% of the variance (R² = 0.62). The derived regression equation provides a practical tool for estimating age based on clinical examinations alone. However, the moderate R² value observed in the present study suggests that, while eruption status is a strong predictor, it should ideally be used in conjunction with other indicators to improve precision. Tandon et al. evaluated an India-specific regression formula for dental age estimation and found it to be reasonably reliable for the local population in and around Bahadurgarh and Haryana [10]. Their findings highlight the importance of developing and validating population-specific models to improve forensic age estimation accuracy. Sarkar et al. compared two dental age estimation formulas in an Indian population and reported variability in the accuracy between the methods [11]. Their findings emphasize the need for population-specific validation and careful selection of appropriate formulas for reliable forensic age estimation.

A meta-analysis by Prasad and Kala evaluated the accuracy of commonly used dental age estimation methods in the Indian population and found considerable variability among techniques [12]. Their findings underscore the importance of population-specific validation and suggest that no single method is universally reliable for precise age estimations. The meta-analysis by Esan et al. compared the Demirjian and Willems methods across different populations and found variability in their accuracy depending on population characteristics [13]. Their findings highlight the influence of ethnic and geographic factors and reinforce the need for population-specific calibration of dental-age estimation methods.

An important observation in this study was the absence of statistically significant differences in eruption patterns between the male and female participants. This suggests that within the studied age range, tooth eruption follows a similar trajectory across sexes, supporting the use of a unified predictive model. This finding aligns with those of Demirjian et al., who reported minimal sex-based differences in certain dental developmental parameters, although some studies have suggested a slightly earlier eruption in females [14]. The lack of significant sex variation in the present study may reflect population-specific characteristics or the age ranges examined. Sun et al. reported that both sex and the timing of the mixed dentition phase significantly influenced discrepancies between chronological and dental ages in children [15]. Their findings highlight that variations in eruption timing can affect age estimation accuracy, emphasizing the need to consider developmental stages and population-specific factors.

The graphical analysis further reinforced the statistical findings. The scatter plot demonstrated a clear linear trend with minimal dispersion, indicating good predictive reliability, whereas the age-wise distribution of eruption status highlighted the biological progression from unerupted to fully erupted teeth with increasing age. These visual representations strengthen the validity of eruption-based assessments as practical tools in both clinical and forensic settings.

From clinical and forensic perspectives, the findings of this study have important implications. Tooth eruption status offers a simple, noninvasive, and cost-effective method for age estimation that does not require radiographic exposure or specialized equipment. This makes it particularly useful in field conditions, mass disaster scenarios, and resource-limited settings, where radiographic methods may not be feasible. Additionally, the use of a cumulative eruption score provides a quantitative approach that enhances objectivity and reproducibility compared with qualitative assessment methods. In pediatric dentistry, such data can also aid treatment planning, growth assessment, and monitoring of developmental milestones.

Despite its strengths, this study has certain limitations that must be acknowledged. First, the use of convenience sampling may introduce selection bias and limit the generalizability of the findings to a broader population. Second, the cross-sectional design did not account for individual longitudinal variations in eruption timing. Third, potential confounding factors, such as nutritional status, socioeconomic background, and genetic influences, were not controlled, all of which are known to affect dental development. Fourth, although practical, the eruption scoring system is relatively simplified and may not capture subtle variations in the eruption stages. Finally, although statistically significant, the regression model explained only a moderate proportion of the variance, indicating that additional biological markers may be required for more precise age estimation.

Future research should focus on larger multicenter studies using probabilistic sampling methods to enhance generalizability. The incorporation of radiographic parameters, skeletal maturity indicators, and advanced statistical or machine learning models may further improve predictive accuracy. Development of population-specific standards is essential for refining forensic age estimation methods.

## Conclusions

The present study demonstrated a strong and statistically significant relationship between cumulative tooth eruption status and chronological age in children aged 6-13 years. The eruption score showed good discriminatory ability across age groups and served as a reliable predictor of age, with no significant sex differences observed. The derived regression model provides a simple, noninvasive, and clinically applicable tool for age estimation. Although the predictive accuracy was moderate, the method was effective for practical use, particularly in resource-limited and forensic settings. Overall, cumulative eruption scoring can be considered a useful adjunct for age estimation in the pediatric population.
